# Supplementary material for: Read Count Patterns and Detection of Cancerous Copy Number Alterations in Plasma Cell-Free DNA Whole Exome Sequencing Data for Advanced Non-Small Cell Lung Cancer
Source: Int J Mol Sci. 2022 Oct 26;23(21):12932. doi: 10.3390/ijms232112932 (PMC9659213; doi:10.3390/ijms232112932)
Supplement: Supplementary file 1 [file ijms-23-12932-s001.zip › ijms-1953067-supplementary.pdf]

Supplementary material for “Read count patterns and detection of cancerous copy number alterations in plasma cell free DNA whole exome sequencing data for advanced non-small cell lung cancer”

Ho Jang[1], Chang Min Choi[2], Seung Hyeun Lee[3], Sung Yong Lee[4], and Mi-Kyung Jeong[5,*]

[1]Korea Medicine Data Division, Korea Institute of Oriental Medicine, Daejeon 34054, Republic of Korea
[2]Department of Pulmonary and Critical Care Medicine, Department of Oncology, Medical Center, College of Medicine, University of Ulsan, Seoul 05505, Republic of Korea
[3]Division of Pulmonary and Critical Care Medicine, Department of Internal Medicine, Kyung Hee University Medical Center, Kyung Hee University School of Medicine, Seoul 02447, Republic of Korea
[4]Division of Pulmonary, Allergy, and Critical Care Medicine, Department of Internal Medicine, Korea University Guro Hospital, Seoul 08308, Republic of Korea
[5]Korea Medicine Convergence Research Division, Korea Institute of Oriental Medicine, Daejeon 34054, Republic of Korea
[*]oiny2000@kiom.re.kr

ABSTRACT

It is a supplementary material for the “Read count patterns and detection of cancerous copy number alterations in plasma cell free DNA whole exome sequencing data for advanced non-small cell lung cancer”.

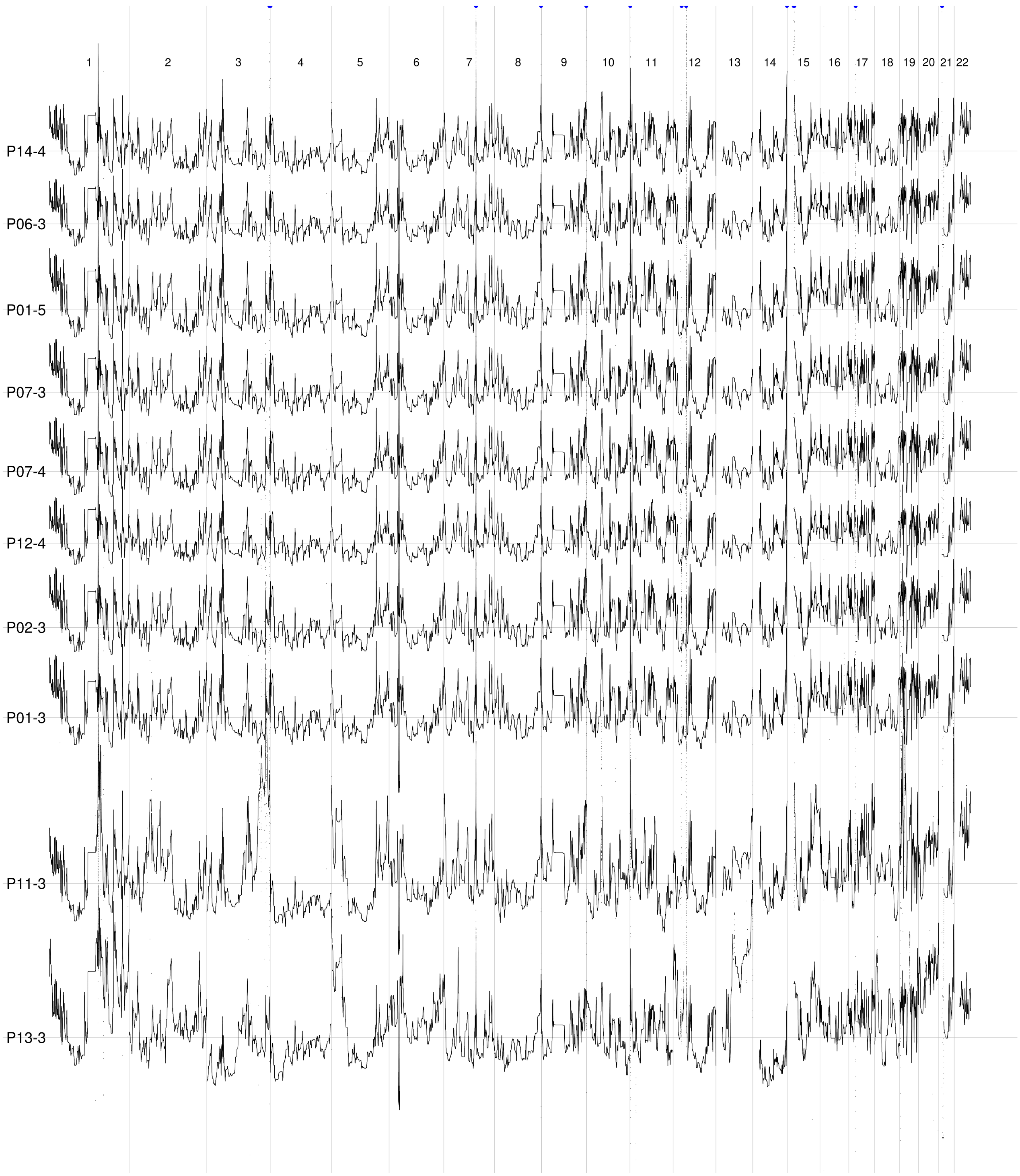

Figure S1. RC patterns of the 2nd WES set

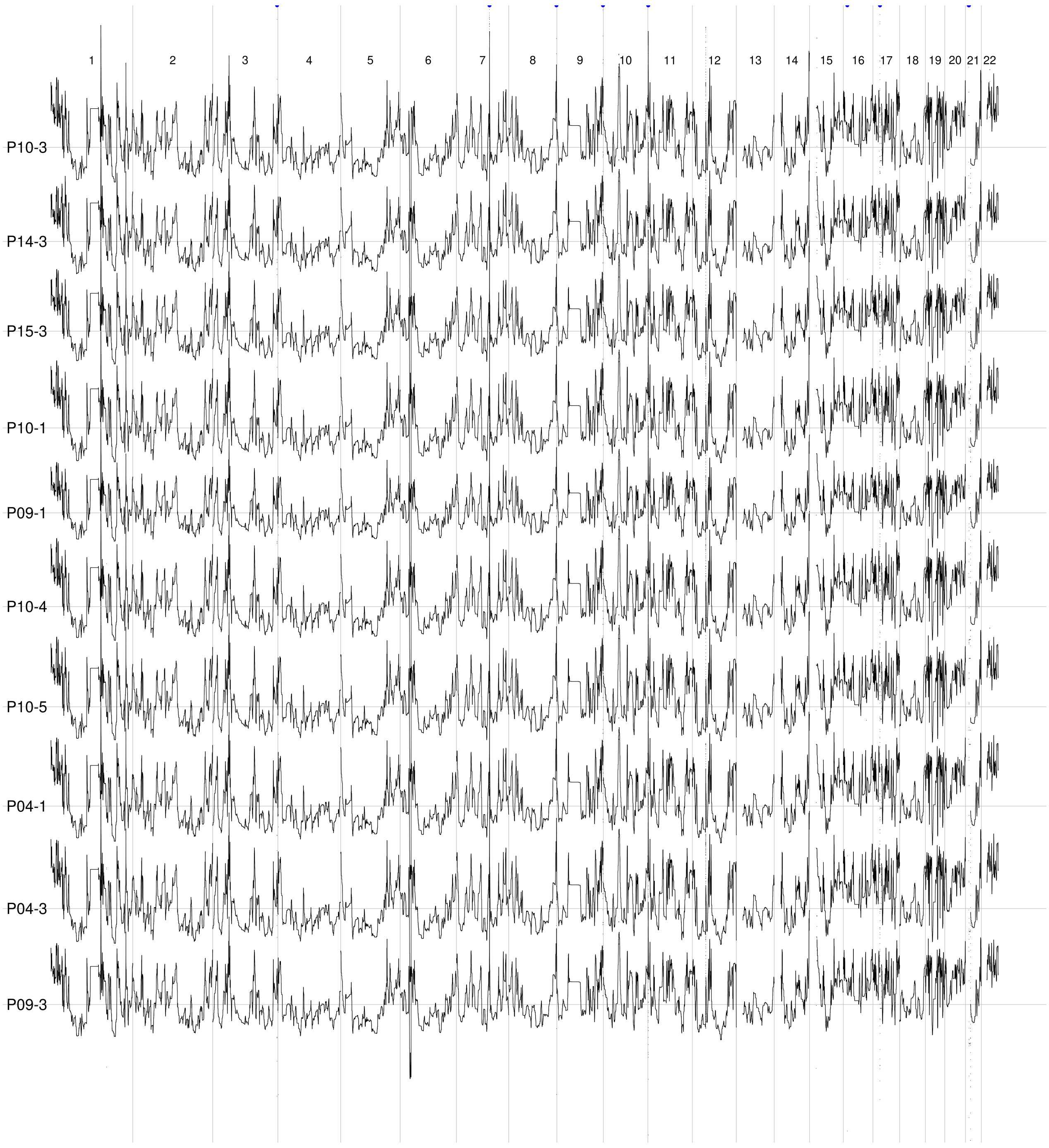

Figure S2. RC patterns of the 3rd WES set (1)

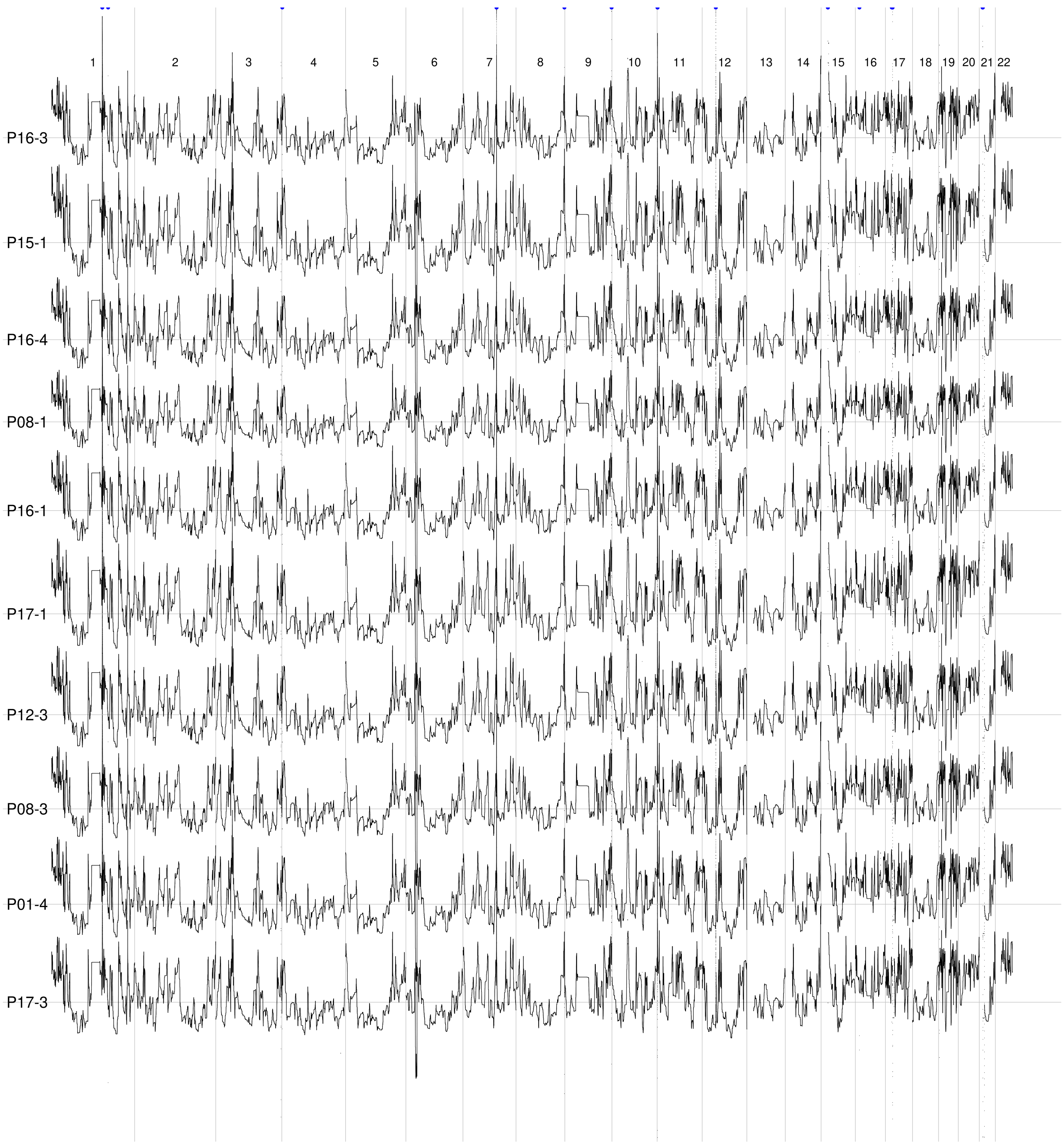

Figure S2. RC patterns of the 3rd WES set (2)

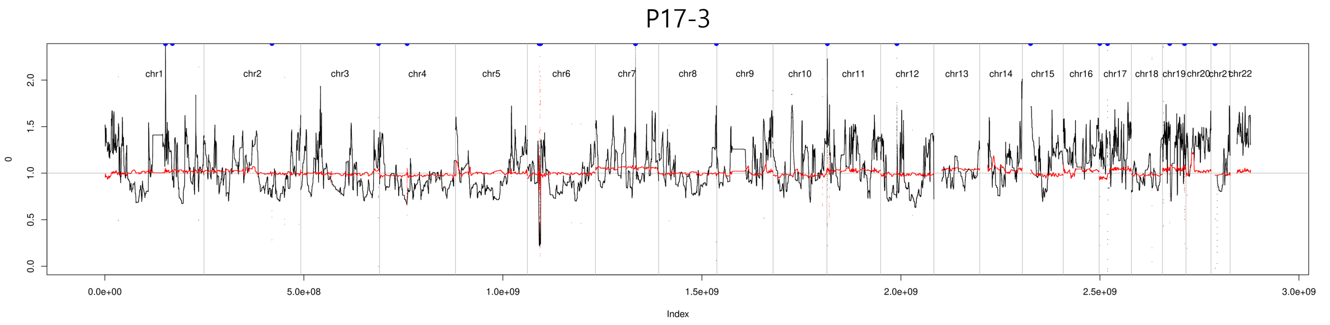

Figure S3. Corrected-RC patterns of P17-3 sample in the 3rd WES set
